# CircFAT1 sponges miR-375 to promote the expression of Yes-associated protein 1 in osteosarcoma cells

**DOI:** 10.1186/s12943-018-0917-7

**Published:** 2018-12-04

**Authors:** Gang Liu, Kangmao Huang, Zhiwei Jie, Yizheng Wu, Junxin Chen, Zizheng Chen, Xiangqian Fang, Shuying Shen

**Affiliations:** 1 Department of Orthopaedic Surgery, Sir Run Run Shaw Hospital, Medical College of Zhejiang University, Sir Run Run Shaw Institute of Clinical Medicine of Zhejiang University, 3 East Qingchun Road, Hangzhou, 310016 Zhejiang Province China; 2 Key Laboratory of Musculoskeletal System Degeneration and Regeneration Translational Research of Zhejiang Province, Sir Run Run Shaw Hospital, Medical College of Zhejiang University, 3 East Qingchun Road, Hangzhou, 310016 Zhejiang Province China

**Keywords:** Osteosarcoma, circFAT1, circular RNA, YAP, miR-375

## Abstract

**Background:**

There is an urgent need to identify new molecular targets for treatment of osteosarcoma. Circular RNAs are a class of endogenous RNAs that are extensively found in mammalian cells and exert critical functions in the regulation of gene expression, but in osteosarcoma the underlying molecular mechanism of circular RNAs remain poorly understood. Here we assessed the tumorigenesis properties of a circular RNA, circFAT1 in osteosarcoma.

**Methods:**

The effects of circFAT1/miR-375/YAP1 was evaluated on human osteosarcoma cells growth, apoptosis, migration, invasion and tumorigenesis. Signaling pathways were analyzed by western blotting, qRT-PCR, fluorescence in situ hybridization, chromogenic in situ hybridization,RNA Binding Protein Immunoprecipitation and immunofluorescence. The consequence of circFAT1 short hairpin RNA combined or not with miR-375 sponge was evaluated in mice bearing 143B xenografts on tumor growth.

**Results:**

In this study, we observed significant upregulation of circFAT1 originating from exon 2 of the FAT1 gene in human osteosarcoma tissues and cell lines. Inhibition of circFAT1 effectively prevented the migration, invasion, and tumorigenesis of osteosarcoma cells in vitro and repressed osteosarcoma growth in vivo. Mechanistic studies revealed that circFAT1 contains a binding site for the microRNA-375 (miR-375) and can abundantly sponge miR-375 to upregulate the expression of Yes-associated protein 1. Moreover, inhibition of miR-375 reversed attenuation of cell proliferation, migration, and invasion, which was induced by circFAT1 knockdown, and therefore promoted tumorigenesis.

**Conclusions:**

Our findings demonstrate a novel function of circFAT1 in tumorigenesis and suggest a new therapeutic target for the treatment of osteosarcoma.

**Electronic supplementary material:**

The online version of this article (10.1186/s12943-018-0917-7) contains supplementary material, which is available to authorized users.

## Background

Osteosarcoma (OS), a common malignant bone cancer, has the highest morbidity in adolescence and childhood [1, 2]. Even with multiple treatments including surgery with chemotherapy, the 5-year survival rate is less than 70% because of tumor recurrence caused by metastasis and drug resistance [3]. Furthermore, efficient therapeutic targets or diagnostic markers for OS have not been identified, making it difficult to improve outcomes. Therefore, it is critical to develop better strategies and new therapeutic methods for avoiding resistance and distant metastasis, thus improving the prognosis of OS.

As a distinct group of noncoding transcripts, circular RNAs (circRNAs), form a closed continuous loop with the 3′RNA and 5′ RNA joined covalently [4–6]. In the past 40 years, circRNAs have been identified in eukaryotic cells by electron microscopy [6] and were previously considered as splicing error by-products. Through the application of high-throughput sequencing and bioinformatics, circRNAs have been successively identified in multiple cell lines and various species [7–11]. Most circRNAs are formed by exon or intron back-splicing. This process differs from the formation of linear RNAs. Two mechanisms exist for the formation of exonic or exon–intron circRNAs: exon skipping and back-splicing [12–14]. Previous studies reported circRNAs as “miRNA sponges” that play an inhibitory role in miRNA regulation [10, 14]. Known as “miR-7 sponges,” antisense to the cerebellar degeneration-related protein 1 (CDR1as), also known as ciRS-7, is one of the most widely known and effective circRNAs [15, 16]. Its function is to sponge miR-7 with approximately 74 canonical binding sites. Therefore, circRNAs may be critical biological markers in the identification of disease mechanisms and for developing new methods for precise diagnosis and effective treatment. However, the function of circRNAs as miRNA sponges for OS remains unknown.

In this study, we identified and characterized the circRNA circFAT1 (originating from exon 2 of FAT1 gene) [17]. Importantly, we found that circFAT1 RNA, rather than FAT1 mRNA, was significantly upregulated in OS tissues and cell lines. Furthermore, we found that inhibiting circFAT1 expression significantly reduced the invasion, metastasis, and proliferation of OS cells via sponging of miR-375, which downregulated the expression of Yes-associated protein 1 (YAP1).

## Methods

### Ethics

All animal experiments were carried out according to the Guide for the Care and Use of Laboratory Animals published by National Institutes of Health and approved by the Ethics Committee of Sir Run Run Shaw Hospital. All experiments strictly followed the panel’s specific guidelines regarding the care, treatment and euthanasia of animals used in the study.

### Clinical samples

Slices of formalin-fixed and paraffin-embedded primary osteosarcoma and chondroma tissues were obtained from biopsies in each 12 patients before administration of neo-adjuvant chemotherapy. Osteosarcoma, chondroma biopsies were histologically characterized by pathologists according to the criteria defined by the World Health Organization. Written informed consent was obtained from each patient before entering this study, and all study protocols were approved by the Ethics Committee of Sir Run Run Shaw Hospital.

### Materials

Antibodies, mice, and other materials. Information regarding the materials used in this study is included in the Additional file 1: Materials and Methods.

### Cell culture

The human cell line hFOB1.19, human osteosarcoma cell lines MG-63, u2os, SJSA-1, HOS and 143B were purchased from FuHeng Cell Center (Shanghai, China). The OS cell lines were authenticated at the by the ShangHai Biowing Applied Biotechnology Co. Ltd., performing a STR profiling analysis, as described by Capes-Davis and according to the ANSI Standard (ASN-0002) set forth by the ATCC Standards Development Organization. Mycoplasma testing was performed using the Venor GeM Mycoplasma Detection Kit (Minerva Biolabs, Berlin, Germany). Cells were cultured as described in detail in the Additional file 1: Materials section.

### Transfection, and viral infection

All the cell lines Cell culture procedures are described in the Additional file 1: Materials and Methods.

### Xenograft tumorigenesis model

Nude mice (nu/nu, male 3- to 4-week-old) were injected subcutaneously with 5 × 10^6^ 143B stable cells. Tumor volumes were calculated from the length (a) and the width (b) by using the following formula: volume (ml^3^) = ab^2^/2. Five weeks after injection, the animals were sacrificed, and tumors were harvested (measured and weighed) and fixed in 4% paraformaldehyde. Wet tumor weight was calculated as mean weight ± standard deviation (SD) in each group.

### Ago2-binding sites from CLIP data sets

The evidence for Ago2-binding sites was obtained from published online cross-linking immunoprecipitation (CLIP) data sets, which are available from doRiNA (a database of RNA interactions in post-transcriptional regulation, http://dorina.mdc-berlin.de). These data sets include Ago2 HITS-CLIP and PAR-CLIP data from HEK-293 cells and several lymphoma cells. We downloaded the available data sets (http://dorina.mdc-berlin.de) and acquired the Ago2-binding sites of circFAT1 genomic region.

### RNA immunoprecipitation

RIP experiments were performed by using the Magna RIP RNA-Binding Protein Immunoprecipitation Kit (Millipore, Bedford, MA). The Ago-RIP assay was conducted in HOS cells stably expressing vector or shcircFAT1. We first constructed the lentivirus vectors harbouring vector or shcircFAT1, and established two stable cell lines of HOS. Approximately 1×10^7^ cells were pelleted and re-suspended with an equal pellet volume of RIP Lysis Buffer (about 100 ml) plus protease inhibitors cocktail and RNase inhibitors. The cell lysates (200 μl) were incubated with 5 μg of control rabbit IgG or antibody against Ago2 (Millipore) coated beads with rotation at 4 °C overnight, respectively. After treating with proteinase K buffer, the immunoprecipitated RNAs were extracted by RNeasy MinElute Cleanup Kit (Qiagen) and reversely transcripted using Prime- Script RT Master Mix (TaKaRa). The abundance of circFAT1 level was detected by qRT–PCR assay.

### Luciferase reporter assay

Cells were seeded in 96-well plates at a density of 5×10^3^ cells per well 24 h before transfection. The cells were co-transfected with a mixture of 50 ng firefly luciferase (FL) reporter vectors, 5 ng Renilla luciferase (RL) reporter vectors (pRL-TK), and miRNA mimics at the indicated concentration. The miRNA mimics were obtained from Life Technologies. After 48 h, the luciferase activity was measured with a dual luciferase reporter assay system (Promega, Madison, WI). Each miRNA or NC RNA was co-transfected with RL reporter and FL reporter with or without the circFAT1 3’-UTR. For comparison, the FL activity was first normalized with RL activity. The effect of each miRNA on luciferase reporter with circFAT1 3’-UTR was then normalized with that on luciferase reporter without circFAT1 3’-UTR. Finally, the fold-change was calculated by each miRNA compared with NC.

### RNA in situ hybridization

Cy3-labeled locked nucleic acid miR-375 probes and Alexa Fluor 488-labeled circFAT1 probes were designed and synthesized by RiboBio (Guangzhou, China), and the probes sequences were available upon request. The signals of the probes were detected by Fluorescent In Situ Hybridization Kit (RiboBio, Guangzhou, China) according to the manufacturer’s instructions. The images were acquired on Nikon A1Si Laser Scanning Confocal Microscope (Nikon Instruments Inc, Japan).

### Pull-down assay with biotinylated circFAT1 probe

Pull-down assay was performed as indicated: In brief, 1 × 10^7^ osteosarcoma cells were harvested, lysed, and sonicated. The circFAT1 probe was incubated with C-1 magnetic beads (Life Technologies) at 25°C for 2 h to generate probe-coated beads. The cell lysates were incubated with circFAT1 probe or oligo probe at 4°C overnight. After washing with the wash buffer, the RNA complexes bound to the beads were eluted and extracted with RNeasy Mini Kit (QIAGEN) for RT–PCR or real-time PCR. Biotinylated-circFAT1 probe was designed and synthesized by RiboBio (Guangzhou, China).

### Wound healing assay

HOS and 143B cells were cultured in six-well plates and scraped with the tip of 200 μl pipette tips (time 0 h). Cell migration was photographed using 10 high-power fields at 0 and 24 h after injury. Remodeling was measured as diminishing distance across the induced injury, normalized to the 0 h control, and expressed as relative migration.

### Transwell migration and matrigel invasion assays

The migration and matrigel invasion assays were conducted by using transwell chamber (for migration assay) or transwell pre-coated matrigel chamber (for invasion assay) according to the manufacturer’s protocol (BD Science, Bedford, MA, USA). The homogeneous single cell suspensions (5 × 10^4^ cells/well for migration, 1 × 10 [5]/well for invasion) were added to the upper chambers and incubated for 24 h. The migration and invasion rates were quantified by counting the migratory and invasive cells at least three random fields.

### Anoikis assay

For anoikis analysis, cells were cultured in suspension for 48 h. Cells were then trypsinized and stained with Annexin V- FITC –PI and analyzed by flow cytometry using the Annexin V-FITC/PI Apoptosis Detection kit (BD Biosciences) following the manufacturer’s instructions. Data were collected on a BD FACSCanto and analyzed using FlowJo software.

### Other in vitro experiments

We performed in vitro experiments as described, including CCK-8 assay, cell transfection and colony formation [18], western blotting [19], flow cytometry, IHC and immunofluorescence microscopy [18, 20], real-time PCR using U6 as an internal control [20], and northern blotting [10, 14]. Given the different sizes of circFAT1 and linear FAT1 mRNA, the samples were separated on a 1.5% formaldehyde agarose gel for circFAT1 and a 0.6% gel for FAT1 mRNA by using voltages of 50 and 90 V, respectively. Tumor-formation assay was described previously [18]. Further details are provided in the Additional file 1: Materials and Methods.

### Statistical analysis

Statistical analyses were performed with 22.0 SPSS. Data are represented as means with SDs, and statistical significance was determined with unpaired Student’s t tests, unless indicated otherwise. P values less than 0.05 were considered statistically significant. Tumor sizes were measured daily to observe dynamic changes in tumor growth and calculated by a standard formula:

## Results

CircFAT1 is abundantly expressed in OS tissues and cell lines and predominantly localized in the cytoplasm.

**Fig. 1:**
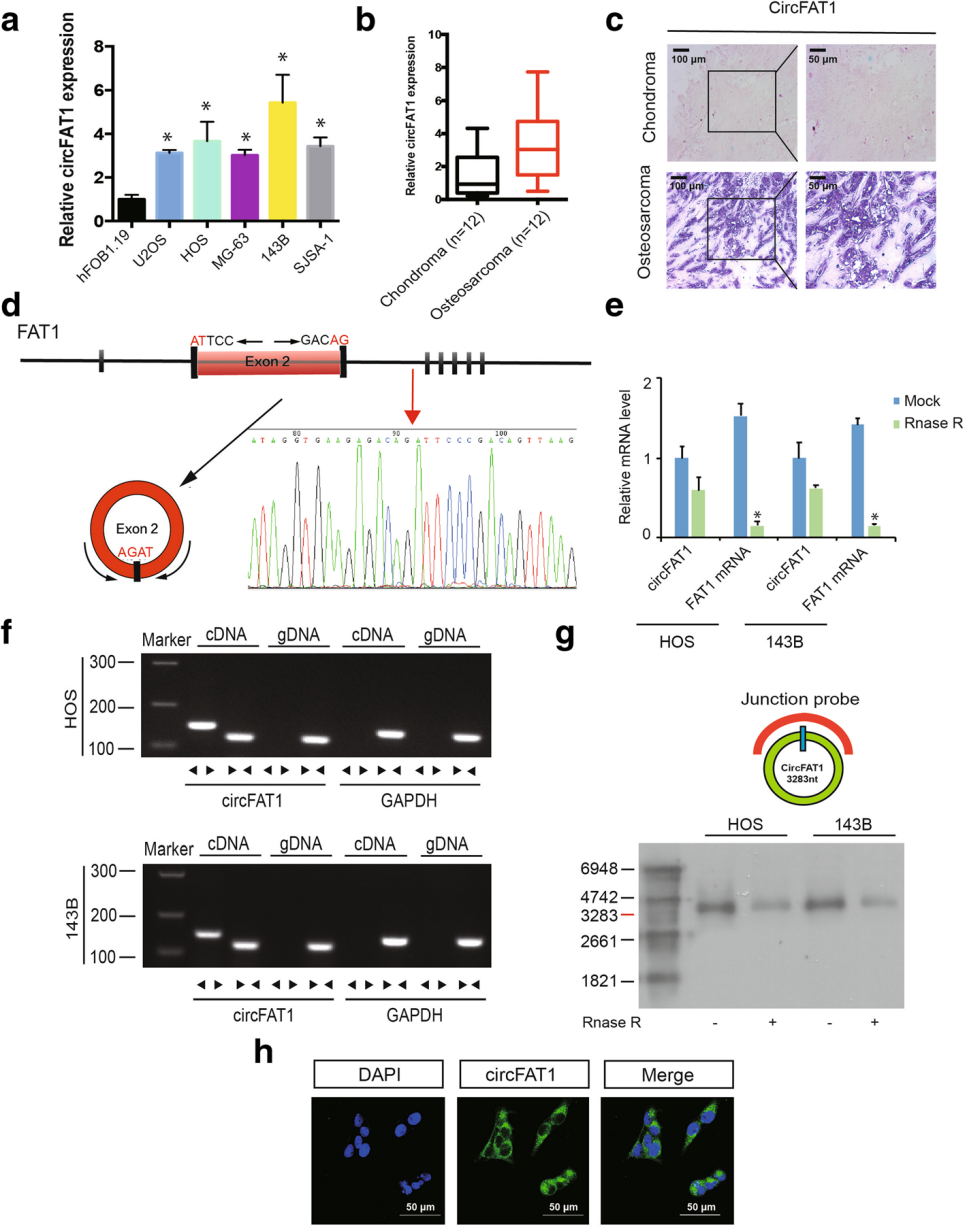
CircFAT1 validation and expression in osteosarcoma tissue and cells. **a** CircFAT1 expression in hFOB1.19 and osteosarcoma (OS) cell lines (OS-732, 143B, HOS, SJSA-1, and MG-63) was evaluated by qRT-PCR. Data represents the mean ± standard deviation (SD) (n = 3). * *P* < 0.05 (**b**) circFAT1 expression was higher in human OS than in chondroma tissue. Data represents the mean ± SD (*n* = 12). **c** CircFAT1 expression was higher in human OS than in chondroma tissue. Representative images are shown (400× magnification). **d** Schematic illustration showed the FAT1 exon 2 circularization forming circFAT1 (black arrow). The presence of circFAT1 was validated by RT–PCR, followed by Sanger sequencing. Red arrow represents “head-to-tail” circFAT1 splicing sites. **e** The expression of circFAT1 and FAT1 mRNA in HOS and 143B cells treated with or without RNase R was detected by real-time PCR. The relative levels of circFAT1 and FAT1 mRNA were normalized to the value measured in the mock treatment. Data represents the mean ± SD (*n* = 3). * P < 0.05. **f** The presence of circFAT1 was validated in HOS and 143B osteosarcoma cell lines by RT–PCR. Divergent primers amplified circFAT1 in cDNA, but not in genomic DNA. GAPDH was used as a negative control. **g** Northern blots for detecting circFAT1 in HOS and 143B cells treated with or without RNase R digestion. The upper panels show the probed blots of circFAT1, and the red triangle represents circFAT1 band size (3283 base pairs (bp)). The lower panels show the gel electrophoretic results of RNA with or without RNase R digestion. **h** RNA fluorescence *in situ* hybridization (FISH) showed that circFAT1 was predominantly localized in the cytoplasm. CircFAT1 probes were labeled with Alexa Fluor 488, Nuclei were stained with DAPI. Scale bar, 50 μm

To investigate the function of circFAT1 in OS development, we detected the expression level of circFAT1 in 12 pairs of chondroma and OS tissues and in OS cell lines. Using reverse transcription-polymerase chain reaction (RT–PCR) and chromogenic in situ hybridization (CISH), we found that circFAT1 was upregulated in OS tissue and cell lines (Fig. 1a–c). We then confirmed head-to-tail splicing in the circFAT1 RT–PCR product, along with the circFAT1 size by Sanger sequencing (Fig. 1d). However, head-to-tail splicing may be produced by trans-splicing or genomic rearrangements. Thus, we ruled out these possibilities as previously described [10, 21]. First, we designed convergent primers to amplify FAT1 mRNA and divergent primers to amplify circFAT1. Using real-time PCR, we confirmed that circFAT1 was resistant to RNase R, while FAT1 mRNA was significantly reduced after RNase R treatment (Fig. 1e). Using cDNA and genomic DNA from the HOS and 143B cell lines as templates, circFAT1 was only amplified by divergent primers in cDNA. We did not detect an amplification product for genomic DNA (Fig. 1f). Second, we performed northern blot analysis for circFAT1 with total RNA extracted from HOS and 143B cells (Fig. 1g). As shown in Figure 1g, both cell lines (with or without RNase R digestion) showed a band of the expected size (3200 base pairs (bp)) using a digoxigenin-labeled circFAT1-specific probe targeting the junction region. RNA fluorescence in situ hybridization (FISH) assay demonstrated that circFAT1 was predominately localized in the cytoplasm (Fig. 1h).

### Knockdown of circFAT1 inhibits migration and invasion of OS cells in vitro

**Fig. 2:**
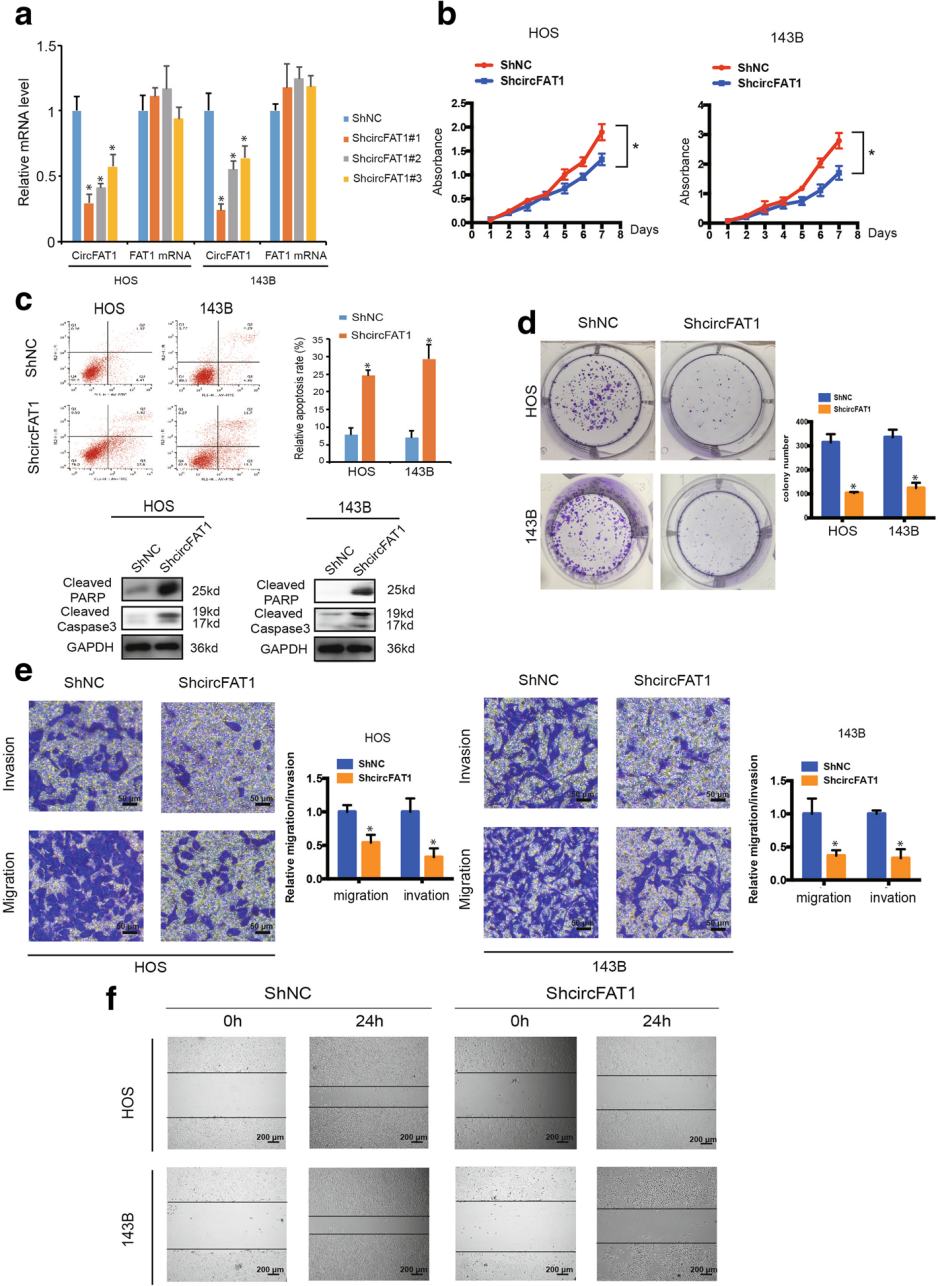
Knockdown of circFAT1 inhibits the migration and invasion of osteosarcoma cell lines *in vitro*. **a** The expression levels of circFAT1 and FAT1 mRNA in HOS and 143B cells after stable transfection of circFAT1 short hairpin RNAs or vector plasmids were detected by real-time PCR. Data represents the mean ± SD (*n* = 3). * P < 0.05. **b** ShRNA-mediated circFAT1 knockdown suppressed OS cell proliferation, as determined in the CCK-8 assay. Data represents the mean ± SD (n = 6). **c** HOS and 143B cells were transfected with shcircFAT1, followed by Annexin V-FITC/PI staining. The percentage of apoptotic cells is shown as the mean ± S.D. from the three independent experiments. * *P* < 0.05, significantly different compared with the vector group. **d** CircFAT1 knockdown suppresses cell growth, as determined by the colony formation assay (details are shown in the insets). Error bars represent the mean ± SD of three independent experiments. * *P* < 0.05. **e** Cell migration and invasion abilities of HOS and 143B cells transfected with shcircFAT1 or vector were evaluated by Transwell migration and Matrigel invasion assays. Data represents the mean ± SD (*n* = 3). * *P* < 0.05. Scale bar, 50 μm. **f** The effect of shcircFAT1 on cell migration capability was evaluated by the wound healing assay in HOS and 143B cells. Scale bar, 200 μm

To explore the function of circFAT1 in OS cells, we transfected circFAT1 small hairpin RNA (shRNA) constructs into 143B and HOS cells. This transfection targeted the junction sites of circFAT1 and established stable knockdown cells. The expression of circFAT1 was significantly reduced in these cells (Fig. 2a). In contrast, the expression of FAT1 mRNA did not change (Fig. 2a). Accordingly, the proliferation capabilities of OS cells decreased upon transfection with circFAT1 shRNA (shcircFAT1#1) (Fig. 2b). Flow cytometric analysis was conducted to determine the effect of circFAT1 knockdown on the apoptosis rate of OS cells at 48 h post-transfection. As a result, circFAT1 knockdown markedly enhanced OS cell apoptosis, and protein levels of cleaved caspase3 and cleaved PARP (Fig. 2c). Moreover, as shown in Fig. 2d, compared with control cells, circFAT1 knockdown cells exhibited compromised tumor formation. Knockdown of circFAT1 also suppressed the migration and invasion of OS cell lines in Transwell migration and Matrigel invasion assays (Fig. 2e). Consistently, the wound healing assay demonstrated that circFAT1 silencing significantly inhibited cell migration in HOS and 143B cells (Fig. 2f). Together, these results indicate that circFAT1 is involved in OS cell growth and motility in vitro.

### CircFAT1 abundantly sponges miR-375 in OS cells

**Fig. 3:**
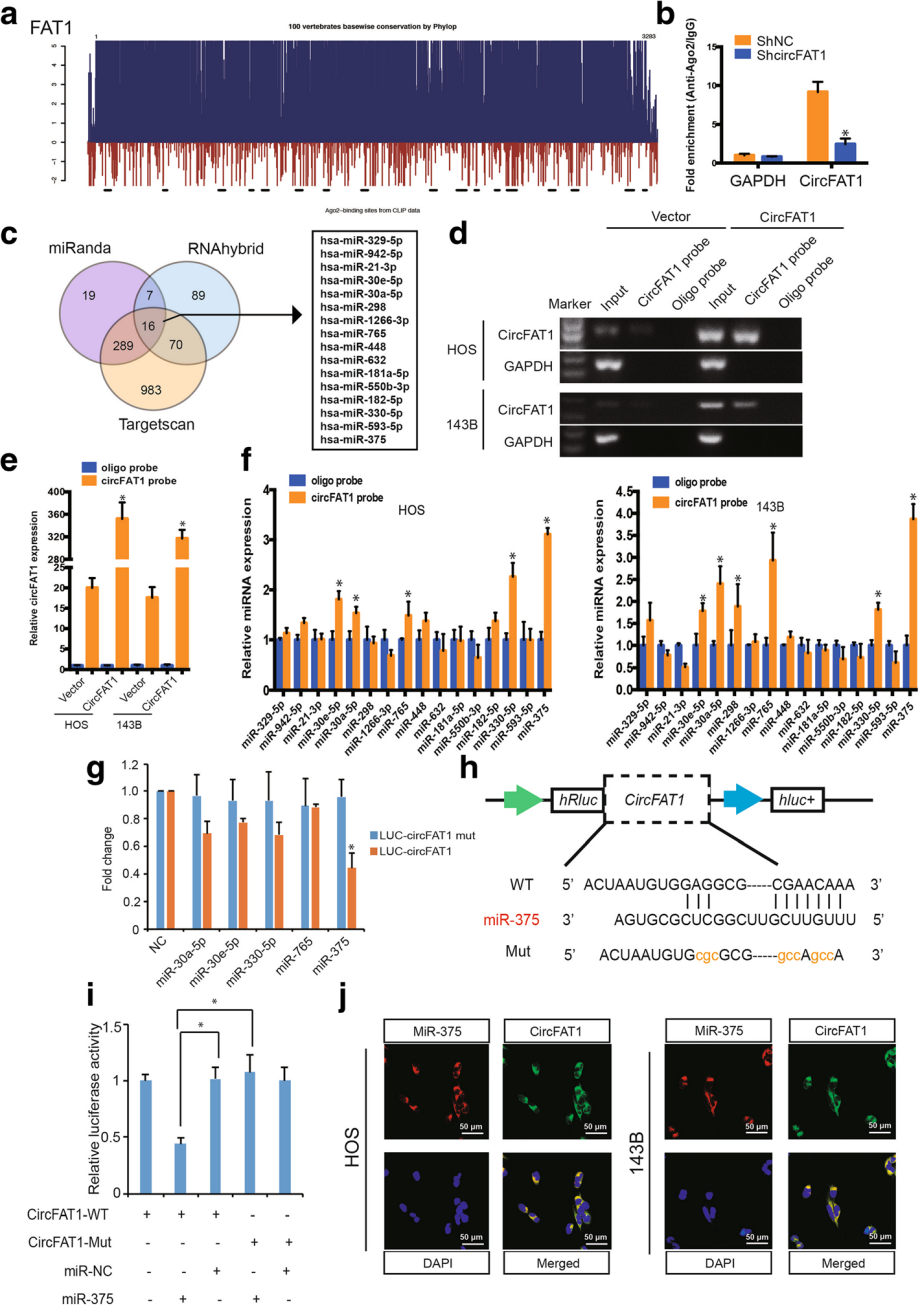
CircFAT1 serves as a sponge for miR-375 in osteosarcoma cells. **a** Schematic illustration showed the conservation across 100 vertebrate species and AGO2 binding sites in the circFAT1 genomic region. **b** AGO2 RNA immunoprecipitation (RIP) assay for circFAT1 levels in HOS cells stably expressing shcircFAT1. Data represents the mean ± SD for three experiments. * *P* < 0.05. **c** Schematic illustration showing overlapping of the target miRNAs of circFAT1 predicted by miRanda, Targetscan, and RNAhybrid. **d**–**e** Lysates prepared from HOS and 143B cells stably transfected with circFAT1 or vector were subjected to RNA pull-down assay and tested by (**d**) RT–PCR and (**e**) real-time PCR. Relative level of circFAT1 was normalized to input. GAPDH was used as a negative control. Data represent the mean ± SD (*n* = 3). * *P* < 0.05 versus oligo probe (Student’s *t*-test). **f** The relative level of 16 miRNA candidates in the HOS and 143B lysates was detected by real-time PCR. **g** Luciferase reporter assay for the luciferase activity of LUC-circFAT1 or LUC-circFAT1-mutant in HEK-293 T cells co-transfected with miRNA mimics. Data represent the mean ± SD for three experiments. * *P* < 0.05. **h** Schematic illustration demonstrates complementary to the miR-375 seed sequence with circFAT1. Lowercase letters indicate mutated nucleotides. **i** 293T cells were co-transfected with miR-375 mimics and a luciferase reporter construct containing wild-type (WT) or mutated circFAT1. Data represent the mean ± SD (*n* = 3). * *P* < 0.01. **j** Fluorescence *in situ* hybridization (FISH) showing co-localization between circFAT1 and miR-375 in HOS and 143B cells. CircFAT1 probes were labeled with Alexa Fluor 488. Locked nucleic acid miR-375 probes were labeled with Cy3. Nuclei were stained with DAPI. Scale bar, 50 μm

It has been reported that circRNA functions as miRNA sponge in cancer cells [10, 14]. Because circFAT1 is abundant and stable in the cytoplasm, we performed an analysis of online AGO2 immunoprecipitation data including high-throughput sequencing from doRiNA. The results revealed a high degree of AGO2 occupancy in the circFAT1 region that is highly conserved across several vertebrate species (Fig. 3a). To validate this result, we conducted RNA immunoprecipitation for AGO2 in HOS cells and found that endogenous circFAT1 pulled-down from AGO2 antibodies was significantly enriched by qRT–PCR analysis compared with circFAT1 stable knockdown cells (Fig. 3b). To confirm whether circFAT1 could sponge miRNAs in OS cells, we selected 16 candidate miRNAs by overlapping the prediction results of miRNA recognition elements in the circFAT1 sequence using miRanda, Targetscan, and RNAhybrid (Fig. 3c). Next, we investigated whether circFAT1 could directly bind these candidate miRNAs. We designed a biotin-labeled circFAT1 probe and verified the pull-down of circFAT1 in OS cell lines. We also found that pull-down efficiency was significantly enhanced in circFAT1-overexpressing stable cells (Fig. 3d and e). The levels of the 16 candidate miRNAs were determined using a pull-down assay and detected by real-time PCR. As shown in Fig. 3f, five microRNAs were pulled-down by circFAT1 in both HOS and 143B cells. We then mutated each of the five miRNA predicted target sites using a luciferase reporter including circFAT1 in 3′- untranslated region (3′-UTR). Compared with the control, three miRNAs (miR-30a, miR-330, and miR-375) reduced the luciferase reporter activities by at least 25% (Fig. 3g). Transfection of the three miRNAs had no significant effect on luciferase activity when the corresponding target sites were mutated from the luciferase reporter. Because miR-375 decreased luciferase activity to the greatest extent and circFAT1 contained an miR-375 8mer-1a binding site (Fig. 3h), we transfected miR-375 mimics into HEK-293T cells and observed that the luciferase activities of WT reporter was significantly reduced when compared with the Mut reporter (Fig. 3i). Supporting this result, the RNA FISH assay revealed that circFAT1 interacts with miR-375 in the cytoplasm of OS cells (Fig. 3j) and tissues (Additional file 2: Figure S3). These results suggest that circFAT1 functions as a sponge of miR-375.

### miR-375 is downregulated in OS tissues and cell lines and inhibits cell migration, invasion, and proliferation by targeting YAP1 in vitro

**Fig. 4:**
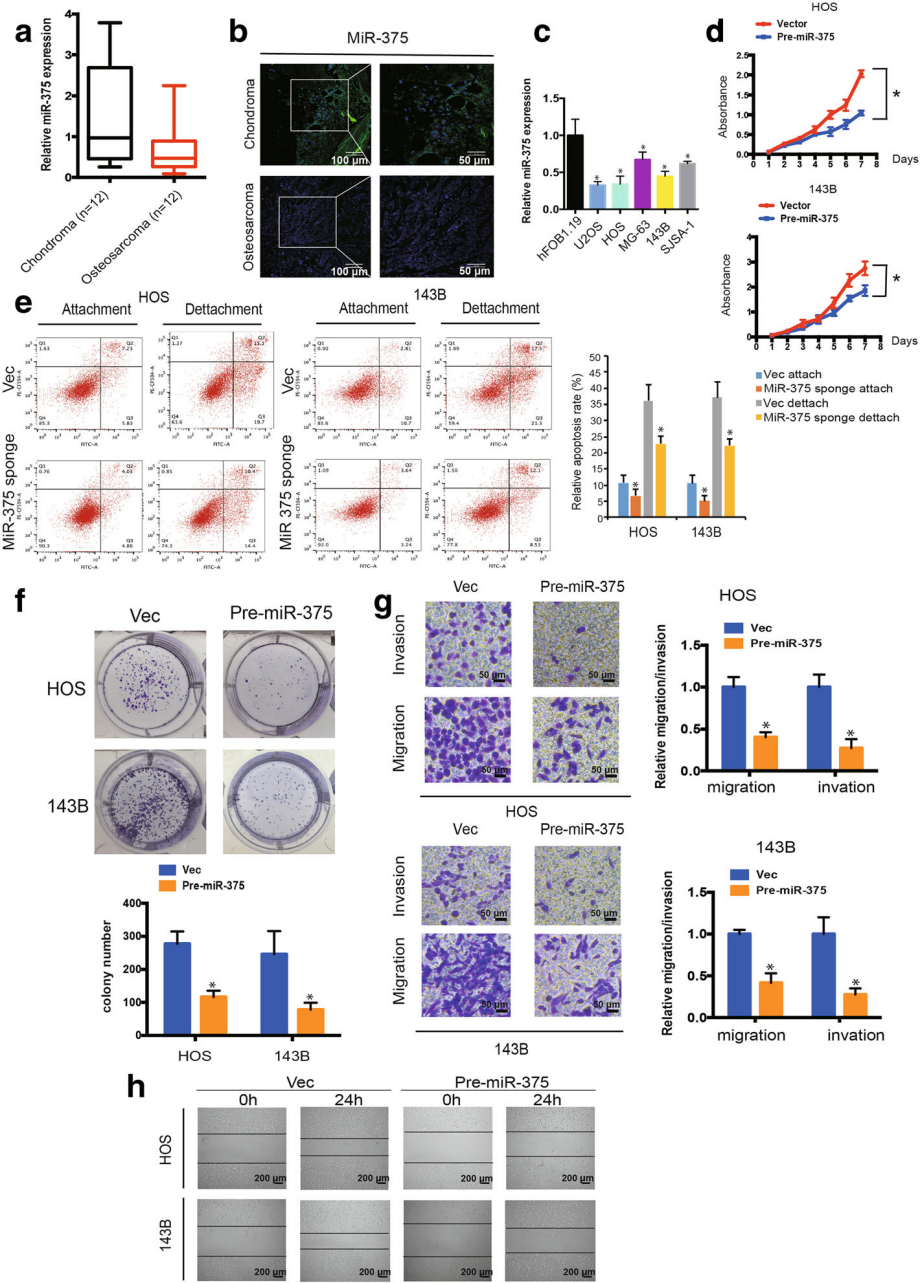
MiR-375 is associated with OS cell migration and invasion. **a** miR-375 expression was lower in human OS than in chondroma tissue. Data represent the mean ± SD (*n* = 12). **b** miR-375 expression was lower in human OS than in chondroma tissue. Representative images are shown (Scale bars = 100 μm or 50μm). **c** miR-375 expression in hFOB1.19 and OS cell lines (OS-732, 143B, HOS, SJSA-1, and MG-63) was evaluated by qRT-PCR. Data represents the mean ± SD (*n* =3). * *P* < 0.05 (**d**) Pre-miR-375-mediated miR-375 overexpression suppressed OS cell proliferation, as determined by the CCK-8 assay. Data are presented as the mean ± SD (*n* = 6). **e** HOS and 143B cells were transfected with miR-375 sponge, followed by Annexin V-FITC/PI staining. The percentage of apoptosis cells is shown as the mean ± SD from three independent experiments. * *P* < 0.05, significantly different compared with the vector group. **f** miR-375 overexpression suppresses cell growth, as determined by the colony formation assay (details are shown in the insets). Error bars represent the mean ± SD of three independent experiments. * *P* < 0.05. **g** Cell migration and invasion of HOS and 143B cells, transfected with pre-miR-375 or vector, were evaluated by Transwell migration and Matrigel invasion assays. Data represent mean ± SD (*n* = 3). * *P* < 0.05. Scale bar, 50 μm. **h** The effect of pre-miR-375 on cell migration capability was evaluated by a wound healing assay in HOS and 143B cells, respectively. Data represents the mean ± SD (*n* = 3). * *P* < 0.05. Scale bar, 200 μm

Our previous studies of TAZ confirmed its high expression in human OS [18, 22]. We also found that TAZ increases the migration, invasion, and proliferation of OS cells. Using real-time PCR and FISH, we found that miR-375 was downregulated in human OS tissues compared with chondroma tissues (Fig. 4a and b). miR-375 was also downregulated in OS cells compared with hFOB1.19 cells (Fig. 4c). To investigate the function of miR-375 in OS cell lines, we constructed over-expression of miR-375 in stable cells. We used CCK-8 assays to determine cell viability. The results showed a significant decrease in cell proliferation after upregulation of miR-375 expression (*P* < 0.05; Fig. 4d). Furthermore, we demonstrated that downregulation of miR-375 clearly induced lower levels of anoikis 48 h after transfection of the miR-375 sponge (Fig. 4e, *P* < 0.05). In addition, miR-375 overexpression inhibited colony formation compared with control cells (*P* < 0.05 for both; Fig. 4f). The Transwell assay (Fig. 4g) and wound healing assay (Fig. 4h) showed that overexpression of miR-375 significantly inhibited the migration and invasion of OS cells. These results indicate that miR-375 inhibits the migration, invasion, and proliferation of OS in vitro.

### MiR-375 targets YAP1 in OS

**Fig. 5:**
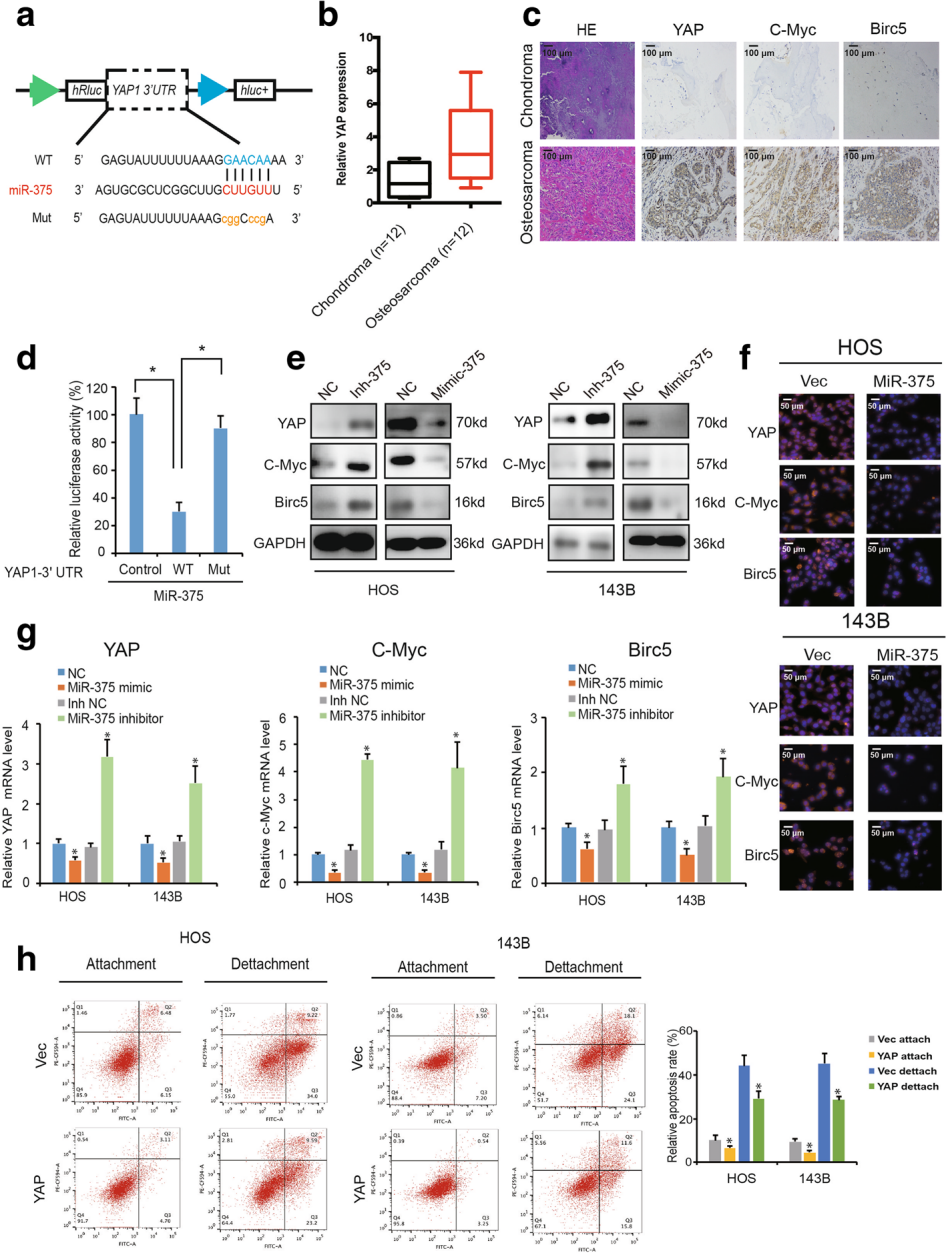
YAP1 is a direct target of miR-375. **a** Schematic illustration showing complementarity to the miR-375 seed sequence in the 3′-UTR of YAP1. Lowercase letters indicate mutated nucleotides. **b** YAP1 expression was higher in human OS than in chondroma tissue. Data represent the mean ± SD (*n* = 12). **c** YAP, c-Myc, and Birc5 expression was higher in human OS than in chondroma tissue. Representative images are shown (Scale bars = 100 μm). **d** 293T cells were co-transfected with pre-miR-375 and a luciferase reporter construct containing wild-type (WT) or mutated YAP1 3′-UTRs. Data represents the mean ± SD (n = 3). * *P* < 0.05. **e**–**f** miR-375 overexpression reduced YAP1, c-Myc, and Birc5 (**e**, **f**) protein and (**g**) mRNA levels while miR-375 inhibition increased YAP1, c-Myc, and Birc5 (**e**, **f**) protein and (**g**) mRNA levels. Cells were transfected with NC or miR-375 mimics/inhibitor, and mRNA or protein levels were evaluated. Protein levels were evaluated by western blot and immunofluorescence, mRNA levels were evaluated by qRT-PCR. Data represent the mean ± SD (*n* = 3). * *P* < 0.05. Scale bars = 50 μm. **h** Overexpression of YAP1 inhibits anoikis in osteosarcoma cells. HOS and 143B stable cells were subjected to suspension culture for 48 h. Anoikis rates were determined by Annexin V-FITC/PI staining. Data represent the mean ± SD (*n* = 3). * *P* < 0.05

To elucidate the function of miR-375 is OS, we used PicTar and TargetScan algorithms to predict potential targets of miR-375. The YAP1 3′-UTR mRNA contained sequences complementary to the miR-375 seed sequence (Fig. 5a). Interestingly, YAP1 mRNA levels were higher in OS than in chondroma tissue (* *P* < 0.01; Fig. 5b). Furthermore, our immunohistochemistry results demonstrated that YAP1 was overexpressed in OS tissues compared with chondroma tissues; the YAP1-targeted genes c-Myc and Birc5 were also overexpressed (Fig. 5c).

To verify whether YAP1 is a direct target of miR-375, we constructed 3′-UTR sensors and co-transfected 293T cells with pre-miR-375. We observed reduced luciferase activity for of the YAP1 3′-UTR in the presence of miR-375 (Fig. 5d). To verify target specificity, we generated mutated forms of the YAP1 3′-UTR, in which the miR-375-binding site was abolished. Co-transfection of pre-miR-375 with the mutant construct abrogated the decrease in wild-type 3′-UTR luciferase activity, indicating that miR-375 specifically regulated YAP1 expression (Fig. 5d). Additionally, transfection of miR-375 mimics reduced endogenous YAP1 protein and mRNA levels, as well as the YAP1-targeted genes c-Myc and Birc5 (Fig. 5e, f and g). However, reducing endogenous miR-375 levels (using miR-375 inhibitor) had the opposite effect in both cell lines (Fig. 5e, f and g). These results indicate that miR-375 targets YAP1 in OS. Consistent with our previous studies [20], YAP1 is important for OS cell apoptosis during anoikis and cell detachment. Overexpression of YAP1 decreased anoikis in both HOS and 143B osteosarcoma cells (Fig. 5h).

**Fig. 6:**
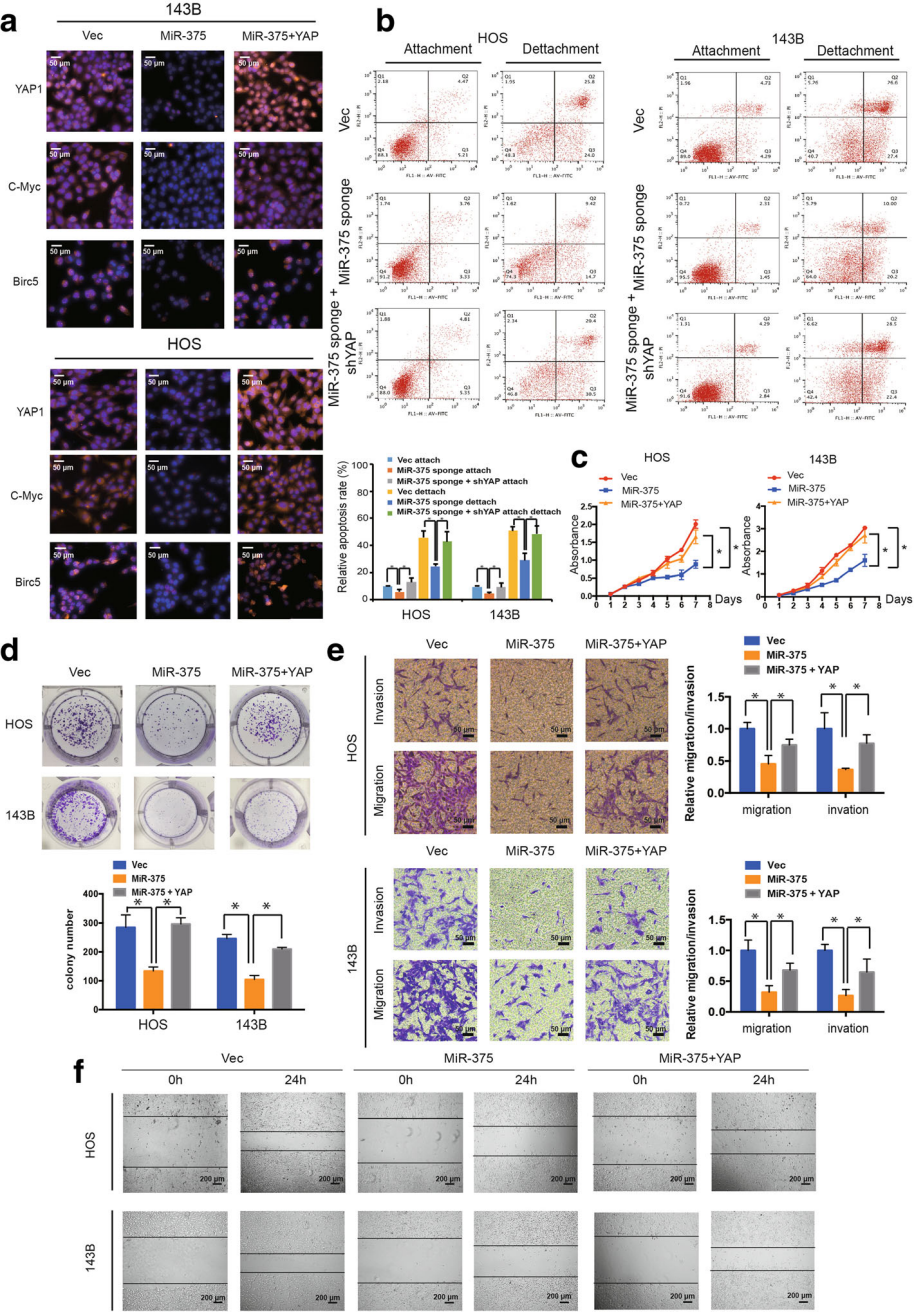
YAP mediates the tumor-suppressive function of miR-375 in OS cells in vitro. **a** The expression of YAP1, c-Myc, and Birc5 in HOS and 143B cells was detected by immunofluorescence analysis. Cells were co-transfected with miR-375 and YAP1 plasmids or control vector. Data represents the mean ± SD (*n* = 3). Scale bars = 50 μm. **b** Downregulation of YAP1 and miR-375 induced more anoikis in osteosarcoma cells compared with miR-375 knockdown alone. HOS and 143B stable cells were subjected to suspension culture for 48 h. Anoikis rates were determined by Annexin V-FITC/PI staining and FACS. Data represents the mean ± SD (*n* = 3). * *P* < 0.05. **c** Proliferation of OS cells, transfected with pre-miR-375 or control vector with or without YAP1, was evaluated by the CCK-8 assay. Data represents the mean ± SD from three independent experiments. **d** YAP1 overexpression stimulated the growth of miR-375 overexpression cells, as determined by the colony formation assays (details are shown in the insets). Data represent the mean ± SD (*n* = 3). * *P* < 0.05. **e** Effects of miR-375 overexpression on cell migration and invasion were abrogated by YAP1 overexpression. Migration and invasion of OS cells co-transfected with pre-miR-375 and YAP or control shRNA were evaluated with the Matrigel and Transwell invasion assays, respectively. Scale bars = 50 μm. **f** The effect of miR-375 and YAP overexpression on cell migration was evaluated by wound healing assay in HOS and 143B cells, respectively. Scale bars = 200 μm

We next investigated whether the tumor-suppressive function of miR-375 is mediated by YAP1 inhibition. Our immunofluorescence results (Fig. 6a) demonstrated that miR-375 overexpression decreased the expression of YAP1, c-Myc, and Birc5, which was rescued by YAP1 overexpression. Remarkably, we found that both YAP1 and miR-375 knockdown augmented OS cell anoikis compared with miR-375 knockdown alone (Fig. 6b). Furthermore, increasing YAP1 levels abrogated the growth inhibition (Fig. 6c) and colony formation (Fig. 6d) induced by miR-375 overexpression, as well as decreased cell migration and invasion of OS cells (Fig. 6e and f). These results support that miR-375 targets YAP1 and mediates the tumor-suppressive function of miR-375.

### Knockdown of miR-375 reverses shcircFAT1-induced attenuation of cell proliferation, migration, and invasion in OS cells

**Fig. 7:**
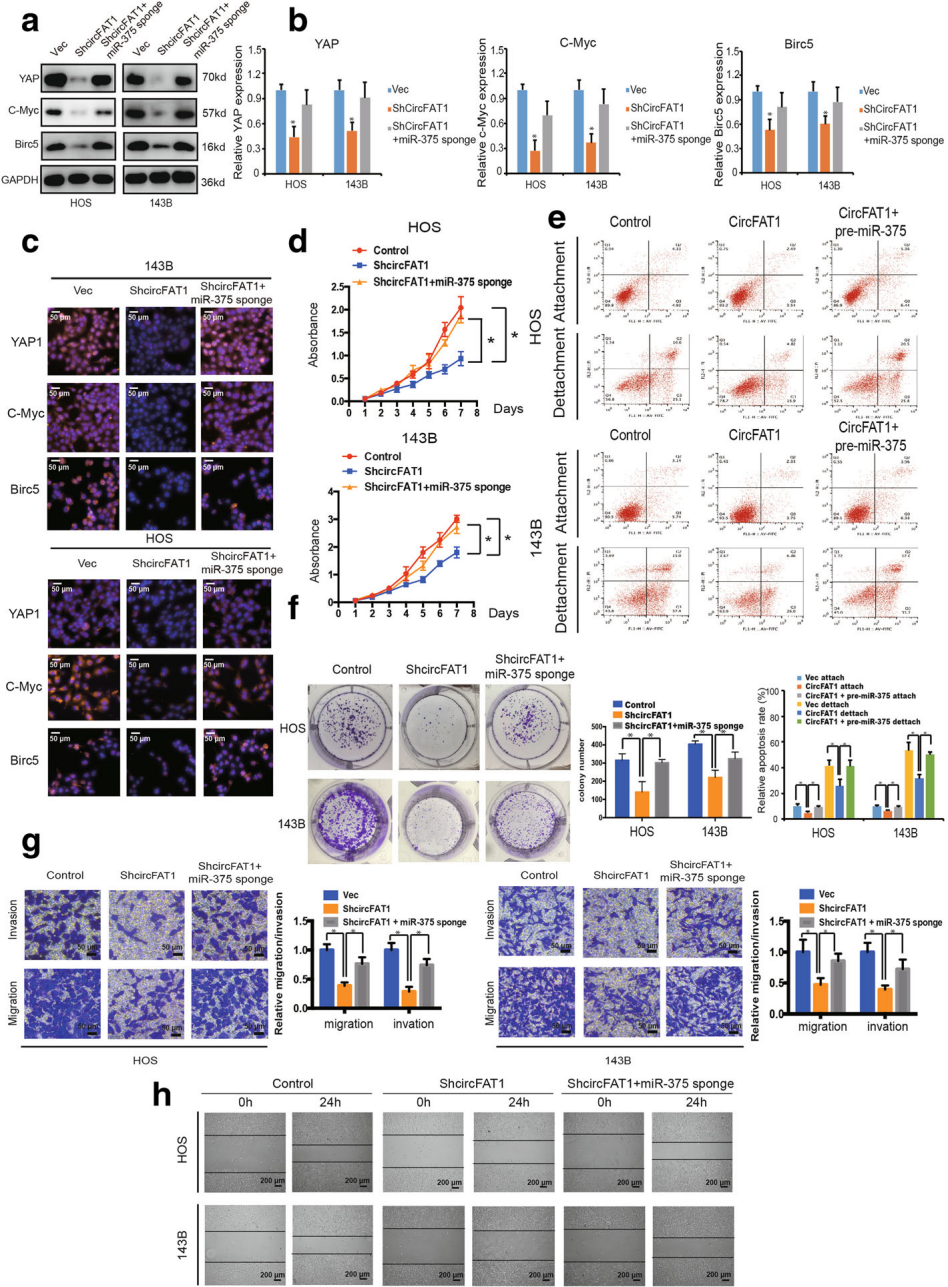
Knockdown of miR-375 reverses shcircFAT1-induced attenuation of cell proliferation, migration, and invasion in OS cells. **a** The protein expression of YAP1, c-Myc, and Birc5 in HOS and 143B cells was detected by western blot analysis. Cells were co-transfected with shcircFAT1 and miR-375 sponge or control vector. Data represent the mean ± SD (*n* = 3). **b** The mRNA expression of YAP1, c-Myc, and Birc5 in HOS and 143B cells was detected by qRT-PCR analysis. Cells were transfected with control vector and shcircFAT1 with or without miR-375 sponge. Data represent the mean ± SD (*n* = 3). * *P* < 0.05 (**c**) The expression of YAP1, c-Myc, and Birc5 in HOS and 143B cells was detected by immunofluorescence analysis. Cells were transfected with control vector and shcircFAT1 with or without miR-375 sponge. Data represent the mean ± SD (*n* = 3). Scale bars = 50 μm. **d** Proliferation of OS cells transfected with control vector and shcircFAT1 with or without miR-375 sponge were evaluated by the CCK-8 assay. Data represents the mean ± SD of three independent experiments. **e** Overexpression of circFAT1 and miR-375 induced more anoikis in osteosarcoma cells compared with circFAT1 overexpression alone. HOS and 143B stable cells were subjected to suspension culture for 48 h. Anoikis rates were determined by Annexin V-FITC/PI staining and FACS. Data represents the mean ± SD (*n* = 3). * *P* < 0.05. **f** miR-375 downregulation stimulated the growth of circFAT1 inhibition cells, as determined by colony formation assays (details are shown in the insets). Data represent the mean ± SD (*n* = 3). * *P* < 0.05. **g** Effects of circFAT1 inhibition on cell migration and invasion were eliminated by miR-375 downregulation. Migration and invasion of OS cells transfected with control vector and shcircFAT1 with or without miR-375 sponge were evaluated by the Matrigel and Transwell invasion assays, respectively. Scale bars = 50 μm. **h** The downregulation of circFAT1 and miR-375 on cell migration capability was evaluated by a wound healing assay in HOS and 143B cells, respectively. Data represent mean ± SD (*n* = 3). * *P* < 0.05. Scale bar, 200 μm

To examine whether circFAT1 promoted OS cell proliferation, migration, and invasion via interacting with miR-375, we co-transfected pre-miR-375 or miR-375 sponge and the circFAT1 overexpression or knockdown construct into OS cells. The results showed that both the protein and mRNA expression of YAP1 and target genes c-Myc and Birc5 significantly increased in OS cells co-transfected with shcircFAT1 plasmids and miR-375 sponge compared with cells transfected with shcircFAT1 alone (Fig. 7a and b). Immunofluorescence analysis confirmed that the expression of YAP1, c-Myc, and Birc5 increased in 143B and HOS cells transfected with shcircFAT1 and miR-375 sponge together when compared with shcircFAT1 alone (Fig. 7c). Knockdown of both miR-375 and circFAT1 resulted in a higher growth rate than in the circFAT1 inhibition group (Fig. 7d). Furthermore, anoikis cells were also upregulated after treatment with pre-miR-375 and circFAT1 co-transfected cells (Fig. 7e). In addition, downregulation of both miR-375 and circFAT1 promoted colony formation when compared with cells transfected with shcircFAT1 alone (*P* < 0.05 for both; Fig. 7f and Additional file 3: Figure S7a-b). In addition, Transwell Matrigel invasion and wound healing assays indicated that OS cells co-transfected with the miR-375 sponge and shcircFAT1 expression construct demonstrated enhanced invasion and migration capabilities when compared with shcircFAT1-transfected cells (Fig. 7g and h). Together, these data suggest that circFAT1 promotes cell migration, invasion, and proliferation via sponging miR-375 and subsequently induces YAP1 expression in vitro.

### CircFAT1 functions as miR-375 sponge to promote tumorigenesis in vivo

**Fig. 8:**
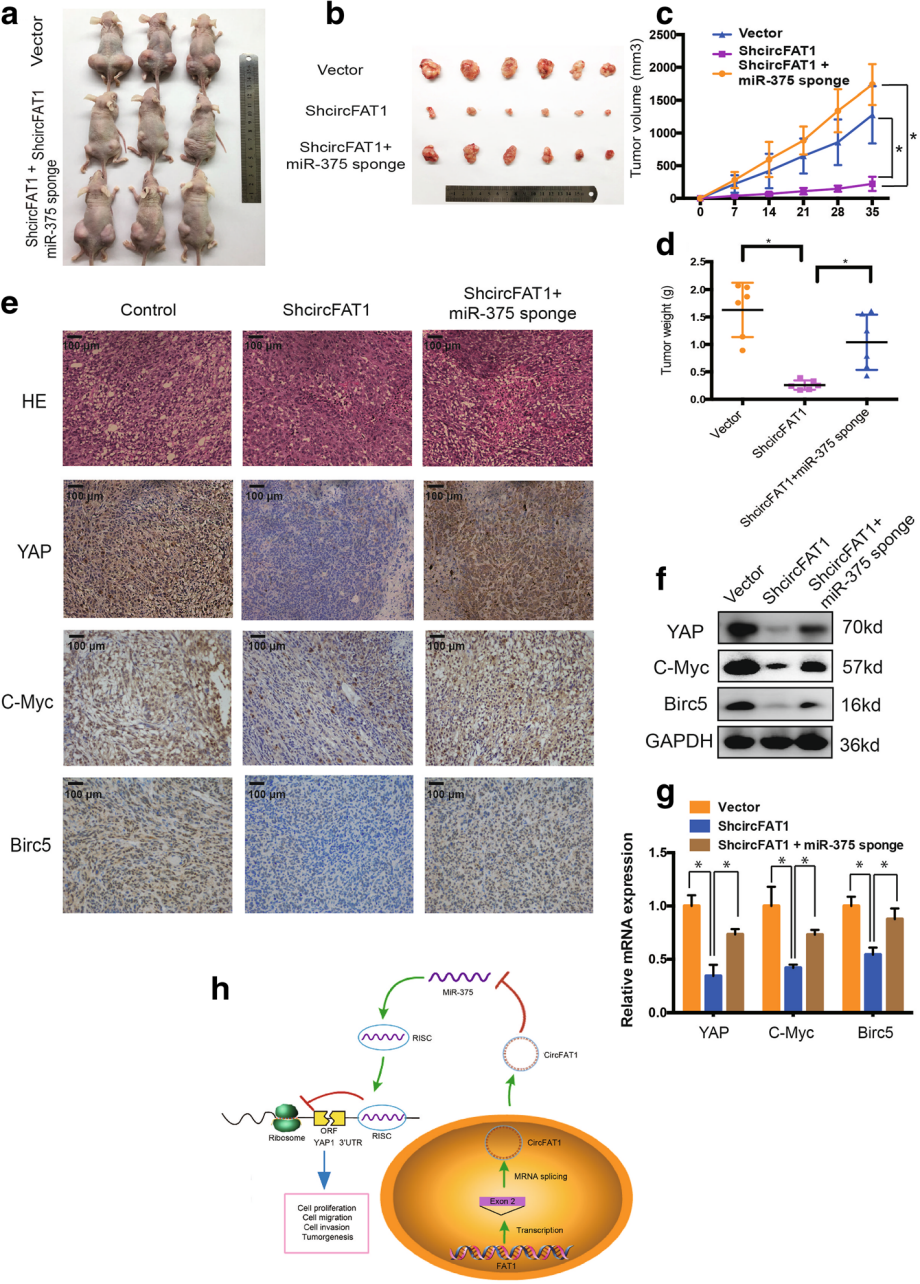
CircFAT1 functions as miR-375 sponge to promote tumorigenesis in vivo. **a**–**b** Nude mice were injected with 5 × 10^6^ 143B stable cells. Tumors were dissected and photographed after 5 weeks. **c** This graph represents tumor volumes (v = ab^2^/2) on the days the mice were injected with control cells or cells transfected with circFAT1 short hairpin (sh)RNA or co-transfected with circFAT1 shRNA and miR-375 sponge (*n* = 6 per group). Data represents the mean ± SD (*n* = 6). **d** Average tumor weight in each group at the end of the experiment (day 35). Data represent the mean ± SD (*n* = 6). * *P* < 0.05. **e** Histological analysis of tumor tissues by hematoxylin and eosin staining. YAP, c-Myc, and Birc5 expression was examined by immunohistochemistry. Scale bars, 100 μm (**f**) Western blot analysis of YAP, c-Myc, and Birc5 in tumors from xenograft mice. **g** qRT-PCR analysis of YAP, c-Myc, and Birc5 expression in tumors from xenograft mice. **h** Schematic illustration of the circFAT1/miR-375/YAP axis

To investigate the effect of circFAT1 and miR-375 in vivo, circFAT1-deficient, miR-375 knockdown, or control 143B cells were subcutaneously injected into nude mice, after which tumor growth was evaluated. Cells lacking both miR-375 and circFAT1 had a higher growth rate than those lacking circFAT1 expression alone (Fig. 8a and b). In contrast, circFAT1 knockdown reduced the volume of 143B-derived tumors in vivo (Fig. 8c), an effect that was abrogated by co-expression with miR-375 sponge constructs. We observed similar results for the average tumor wet weight in the three groups (Fig. 8d).

We next evaluated the relationship between circFAT1 and YAP1 in vivo. circFAT1 knockdown reduced the mean area immunopositive for YAP1, c-Myc, and Birc5 in tumor tissues, as determined by immunohistochemistry (Fig. 8e), this corresponded to a decrease in the levels of YAP1, c-Myc, and Birc5 protein and mRNA (Fig. 8f and g). Inhibition of miR-375 reversed this effect. Interestingly, immunofluorescence (Additional file 4: Figure S5a) and western blot (Additional file 4: Figure S5b) results indicated that the upregulation of c-Myc expression induced by circFAT1 overexpression is independent on Wnt signal pathway. Besides, Kaplan-Meier survival curves of the TCGA sarcoma dataset showed that the patients with high expression of YAP and target genes had a low 10-year overall survival rate, while the patients with high expression of miR-375 had a high 10-year overall survival rate (Additional file 5: Figure S8). These results suggest that circFAT1 sponges miR-375 in OS and miR-375 mediates the tumor-suppressive function of circFAT1 in vivo (Fig. 8h).

## Discussion

In recent years, circRNAs has drawn the increasing attention owning to the development of bioinformatics and high-throughput sequencing technologies [23–26]. With the following distinct characteristics, such as unique structure, conservation across species, cell type-specific and tissue-specific expression, and stable expression in saliva, blood and exosomes [27–29], more than ten thousand different circRNAs have been discovered and studied in various organisms, which also raised the hot topic on circRNAs in malignant tumor research [19, 30, 31]. However, although the studies on circRNAs are being developed on a high speed, there are still many questions unrevealed. In our project, according to the previous studies, we put forward a hypothesis that circRNAs may also have effect on OS progression with the cell type and developmental stage-specific characteristics and can be served as valuable clinical biomarkers for OS.

In the current study, we selected seven tumor-related and highly expressed circRNAs, including circHIPK3, circFOXO3, circRTN4, circITCH, circCdr1as, circFAT1 and circZFR, which have been reported previously to be involved in the progression of colorectal cancer, hepatocellular carcinoma, prostate cancer, gastric cancer and so on. In these seven circRNAs, circHIPK3 has been found to act as a competing endogenous RNA (ceRNA) of miRs with the activity to competitively sequester and quench of miRs, such as miR-7, miR-558 and miR-124, resulting in the dramatic association with the development of a variety of tumors, such as colorectal cancer, hepatocellular carcinoma, and bladder cancer [10, 32, 33]. Besides, circFAT1 and circRTN4 has been showed to be highly expressed in exosomes of colorectal cancer, indicating its potential function in tumorgenesis [34]. CircZFR is another circRNA with significant higher expression in both bladder carcinoma tissues and papillary thyroid carcinoma tissues [35, 36]. What is more, it also has been found that circ-Cdr1as played an important role as a super sponge or competing endogenous RNA (ceRNA) of miR-7, which has the tremendous effects on the growth of many kinds of cancer, such as breast cancer, hepatocellular carcinoma, and cervical cancer [15, 16]. In addition, circFOXO3 and circITCH were reported to suppress tumor cell proliferation, migration and invasion. According to our result, circFAT1 induced the highest rate of apoptosis of osteosarcoma cells compared with other circRNAs (Additional file 6: Figure S1), we therefore choose circFAT1 for the further investigation.

CircFAT1 is derived from the FAT1 gene. Previous studies have demonstrated that the FAT1 gene plays potential roles in tumors and inflammation [37]. The expression of this gene may be a crucial event in cancer invasion and metastasis [38]. In our study, we showed that circFAT1 was highly expressed in osteosarcoma cell lines and tissues. Silencing of circFAT1 inhibits the proliferation, migration and induces apoptosis of osteosarcoma cell line as demonstrated by loss-of-function assays. Previous studies reported that sponge of miRNA is one kind of main mechanisms that circRNAs acts in cancer [15, 16]. Our further studies also revealed that circFAT1 exerts its regulatory functions through harboring miR-375 to rescue the expression of YAP1, a core factor in the Hippo pathway, which is also directly targeted by miR-375.

The Hippo pathway, a highly conserved cell signaling pathway in evolutionary history, plays a critical role in regulating cell proliferation, metastasis, tumorigenesis, and drug resistance in tumors [39–42]. Multiple studies have investigated YAP’s oncogenic role, both in vitro and in vivo [43, 44]. Furthermore, upregulation of YAP gene expression has been detected in cancers such as melanoma, hepatocellular carcinoma, and neurofibromatosis [45–47]. YAP also plays a critical role in the initiation and progression of cancer mediated by other oncogenes such as Kras and beta-catenin [46, 47]. These findings strongly indicate YAP’s essential role in human cancers. For osteosarcoma, YAP/TAZ and its related target gene can be found frequently overexpressed in human OS cells [48, 49]. Reversely, the downregulation of YAP can inhibit the proliferation and migration of OS cell in vitro and impair tumor growth in vivo [48]. On the level of mechanism, some studies showed that NF2 and Kibra, the activators of the Hippo pathway, could upregulate the level of YAP in osteo-tissues mediated by inhibition of Sox2 [48]. The activation of Hedgehog signaling could also trigger the overexpression of YAP via Hippo pathway crosstalk [50]. What is more, the mutation of RASSFs, NF2 and Mob1 tumor suppressor in OS frequently leads to the upregulation of YAP/TAZ [48]. Many important studies have confirmed the critical role of YAP/TAZ in inducing chemo-resistance, radio-resistance even the molecular targeted therapy-resistance in OS [49, 51]. Our previous findings also demonstrated that TAZ, the homologous protein of YAP, is overexpressed in OS and highly related to the proliferation and metastasis of OS cells [20]. Consistently, our current study further indicated that YAP plays a critical role in mediating OS metastasis and tumorigenicity. At the same time, our study revealed a new mechanism of the linkage with circular RNA and the Hippo pathway. Combined with the studies that noncoding RNA plays an important role in the Hippo pathway [52, 53], our study firstly clarified the mechanism that circFAT1 regulates YAP1 expression via sponging miR-375 and acts as an upstream factor in the Hippo/YAP pathway.

## Conclusions

In summary, we characterized and functionally analyzed an abundant circRNA derived from exon 2 of the FAT1 gene. We further demonstrated that circFat1 is upregulated in human OS and can efficiently sponge miR-375 to promote YAP1 expression. We also demonstrate that circFAT1 knockdown effectively inhibited the aggressiveness and metastasis of OS cells by targeting the miR-375/YAP1 axis. Our findings reveal that the circularized protein-coding exons or “sponging miRNAs” have other regulatory effects, and thus provide a new therapeutic target for the treatment of OS.

## Additional files

Additional file 1: Supplementary materials and methods. (DOCX 19 kb)

Additional file 2: **Figure S3.** Co-localization of circFAT1 and miR-375 in osteosarcoma. Fluorescence in situ hybridization (FISH) showing co-localization between circFAT1 and miR-375 in osteosarcoma. CircFAT1 probes were labeled with Alexa Fluor 488. Locked nucleic acid miR-375 probes were labeled with Cy3. Nuclei were stained with DAPI. Scale bar =50 μm. (JPG 958 kb)

Additional file 3: **Figure S7.** Knockdown of miR-375 reverses shcircFAT1-induced attenuation of cell colony formation in OS cells. (a) 143B cells were inoculated in 96-well plates. Typical photos are shown for up to 6 days. Colony formation was monitored for up to 8 days. (b) Single-cell growth curve for up to 8 days. The numbers of cells per well are shown as the inset (Scale bar =100 μm). (JPG 1155 kb)

Additional file 4: **Figure S5.** The upregulation of c-Myc expression induced by circFAT1 overexpression is independent on Wnt signal pathway. (a) The β-catenin subcellular localization is detected by immunofluorescence analysis in OS cells with wnt activators' treatment. Scale bars = 50 μm. (b) The protein expressions of β-catenin and c-Myc in OS cells were detected by western blotting. Cells were co-transfected with circFAT1 or control vector, with or without wnt inhibitors. (JPG 1018 kb)

Additional file 5: **Figure S8.** CircFAT1 expression and Kaplan-Meier survival analysis. (a) QRT-PCR analysis of circFAT1 expression in tumors from xenograft mice. (b) The intra-nuclear localization of c-Myc and YAP. (c) Kaplan-Meier survival analysis of miR-375, YAP1, c-Myc and Birc5 low and high sarcoma patients (log rank test). (JPG 1414 kb)

Additional file 6: **Figure S1.** The apoptosis rate of 143B cells with selected circRNAs knockdown. 143B cells were transfected with siRNAs of selected circRNAs for 48 h. Apoptosis rates were determined by Annexin V-FITC/PI staining. Data represent the mean ± SD (*n* = 3). * *P* < 0.05. (JPG 1341 kb)

## Abbreviations

3′-UTR: 3′- untranslated region
CeRNA: Competing endogenous RNA
CircRNAs: Circular RNAs
OS: osteosarcomaOsteosarcoma
ShRNA: small Small hairpin RNA
YAP1: Yes-associated protein 1
