# Supplementary material for: CircFAT1 sponges miR-375 to promote the expression of Yes-associated protein 1 in osteosarcoma cells
Source: Mol Cancer. 2018 Dec 4;17:170. doi: 10.1186/s12943-018-0917-7 (PMC6280518; doi:10.1186/s12943-018-0917-7)
Supplement: Supplementary file 1 — Supplementary materials and methods. (DOCX 19 kb) [file 12943_2018_917_MOESM1_ESM.docx]

**Supplementary Materials and Methods**

***Antibodies, mice and other materials***

Anti-YAP1, C-Myc, Birc5 antibodies were obtained from Abcam. Anti-GAPDH antibodies were obtained from Cell signaling. Alexa Fluor 488- and Alexa Fluor 594- conjugated secondary antibodies were obtained from Life Technologies. Pre-miR-375 precursor (pre-miR-375) was purchased from Ambion (Foster City, CA, USA). miR-375 sponge was made by cloning a stretch of three perfect match miR-375 antisense oligomer into the LV3 (H1/GFP&Puro) lentiviral vector. SiRNA oligonucleotides toward circFAT1 and control siRNA toward luciferase were purchased from RiboBio. miR- 375 mimics/ inhibitors were obtained from Shanghai GenePharma. Common chemicals were from Sigma or Sangon Biotech.

***Cell culture, transfection, and viral infection***

Human fetal osteoblasts hFOB1.19 was cultured in DMEM-F12 medium (Invitrogen, Karlsruhe, Germany) and Human osteosarcoma cell lines MG-63, HOS, SJSA-1, 143B, U2OS were cultured in RPMI-1640 medium (Invitrogen, Karlsruhe, Germany) supplemented with 10% v/v heat-inactivated fetal bovine serum (Invitrogen) and 1% v/v penicillin/streptomycin (Invitrogen), under adherent conditions at 37 °C in a humidified incubator with 5% CO _2_ /95% air.

Transfection of plasmids was performed using Lipofectamine (Life Technologies) according to the manufacturer’s instructions. Transfection of microRNA mimics/inhibitors or siRNAs was performed using Lipofectamine RNAiMAX (Life Technologies) according to the manufacturer’s instructions.

Lentiviral infection was used to generate various stable cells. Briefly, HEK293T cells were co-transfected with packaging plasmids, as well as viral vectors. 48 hours after transfection, culture medium was supplemented with 5μg/ml polybrene, filtered through a 0.45μm filter, providing for target cells. 36 hours after infection, cells were selected with 2μg/ml puromycin in culture medium.

***CCK-8 assay for cell growth***

For the CCK-8 assay, the cells were seeded in 96-well plates at 2×10^3^ cells/well and incubated for 1 to 7 days. At each time point, CCK-8 (Sigma-Aldrich) was added into each well. After 4 h of incubation, the the absorbance of solution was determined at 450 nm using Versamax microplate reader (Molecular Devices, CA, USA).

***Colony formation assay***

Approximately 1 × 10 ^3^ cells of respective treated HOS or 143B cells were plated in 6-well plates. Cells were fixed with methanol and stained with 0.1% crystal violet after 14 days. Clones containing over 50 cells were counted manually. The experiments were repeated three times to obtain the average colony formation rate.

***Immunohistochemistry***

Surgically resected osteosarcoma and osteochondromas were fixed in neutral buffered formalin for 24 hours at 4°C then embedded and processed according to standard protocols. The sections were deparaffinized through graded ethanol solutions. After an antigen retrieval procedure of 30 min using target retrieval solution (DAKO), the sections were stained with specific antibodies using the avidin-biotin complex system (Vector Laboratory). 3, 3'- diaminobenzidine (DAB) was used as substrate. Cell nuclei were counterstained with Hematoxylin.

***Immunofluorescence***

For YAP1, C-Myc and Birc5 staining, cells were fixed in 4%

paraformaldehyde. Primary antibodies for immunofluorescence microscopy were diluted 1:200-400. Immunofluorescence images were obtained using Nikon Eclipse TI or Zeiss LSM780 confocal microscopes or a Zeiss Colibri epifluorescence microscope and processed with Image J.

***Western blotting***

Western blotting was performed according to protocol. Briefly, cells were first lysed in the protein Lysis Buffer, subjected to SDS-PAGE and then transferred to NC membranes. The membranes were blocked with 5% Nonfat milk and then incubated with antibodies, then washed with TBST. Protein expression was detected by chemiluminescence (ECL, Amersham, Piscataway, NJ). The expression of GAPDH was used as a loading control.

**References**

1 Guo T, Lu Y, Li P *et al*. A novel partner of Scalloped regulates Hippo signaling via antagonizing Scalloped-Yorkie activity. *Cell research* 2013; 23:1201-1214.
2 Zhao B, Ye X, Yu J et al. TEAD mediates YAP-dependent gene induction and growth control. Genes Dev 2008; 22:1962-1971.

3 Ding S, Wu X, Li G, Han M, Zhuang Y, Xu T. Efficient transposition of the piggyback (PB) transposon in mammalian cells and mice. Cell 2005; 122:473-483.
4 Becam I, Rafel N, Hong X, Cohen SM, Milan M. Notch-mediated repression of bantam miRNA contributes to boundary formation in the *Drosophila* wing. *Development* 2011; 138:3781-3789.
5 Brennecke J, Hipfner DR, Stark A, Russell RB, Cohen SM. bantam encodes a developmentally regulated microRNA that controls cell proliferation and regulates the proapoptotic gene hid in *Drosophila*. *Cell* 2003; 113:25-36.
6 Zhang L, Ren F, Zhang Q, Chen Y, Wang B, Jiang J. The TEAD/TEF family of transcription factor Scalloped mediates Hippo signaling in organ size control. *Dev Cell* 2008; 14:377-387.
