# Supplementary figures and images for: CircFAT1 sponges miR-375 to promote the expression of Yes-associated protein 1 in osteosarcoma cells
Source: Mol Cancer. 2018 Dec 4;17:170. doi: 10.1186/s12943-018-0917-7 (PMC6280518; doi:10.1186/s12943-018-0917-7)

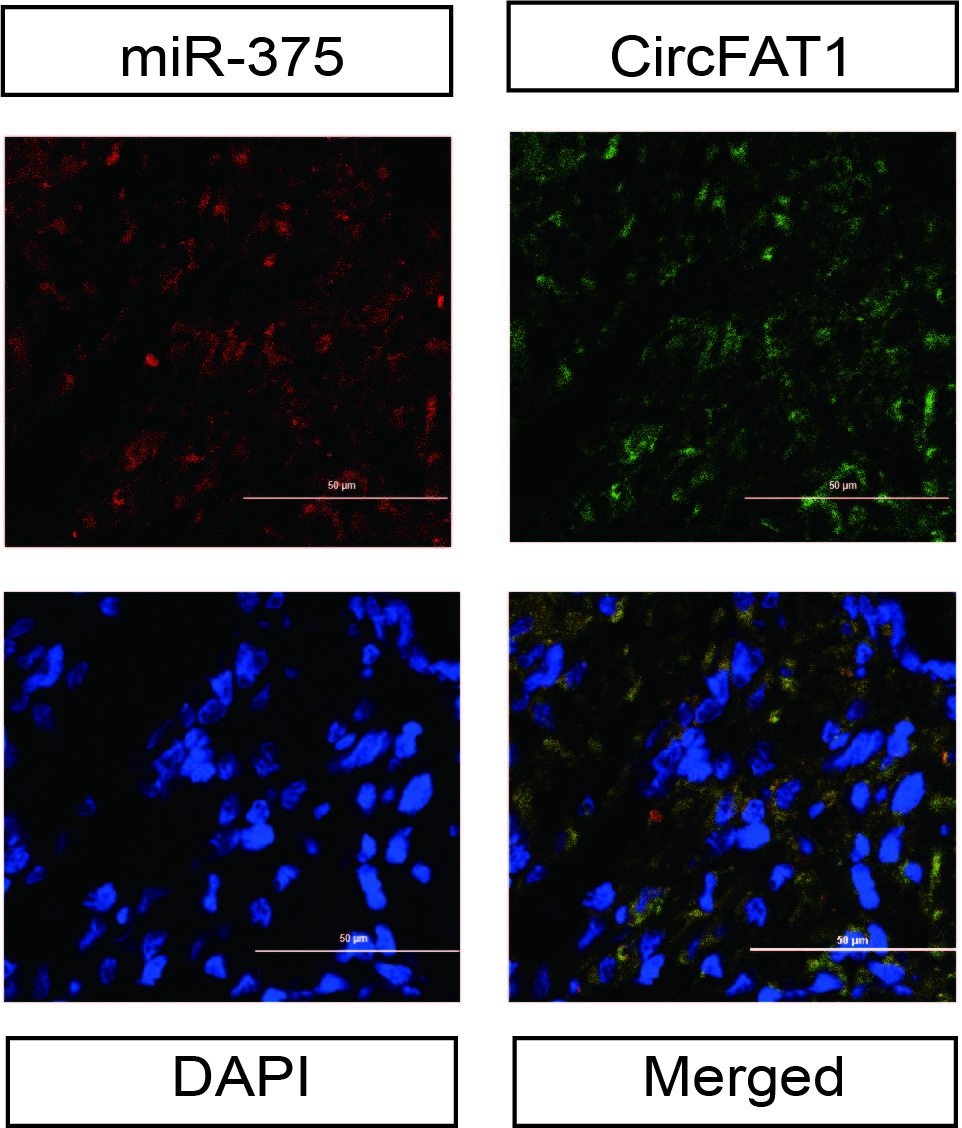

Supplement: Supplementary file 2 — Figure S3. Co-localization of circFAT1 and miR-375 in osteosarcoma. Fluorescence in situ hybridization (FISH) showing co-localization between circFAT1 and miR-375 in osteosarcoma. CircFAT1 probes were labeled with Alexa Fluor 488. Locked nucleic acid miR-375 probes were labeled with Cy3. Nuclei were stained with DAPI. Scale bar =50 μm. (JPG 958 kb) [file 12943_2018_917_MOESM2_ESM.jpg]

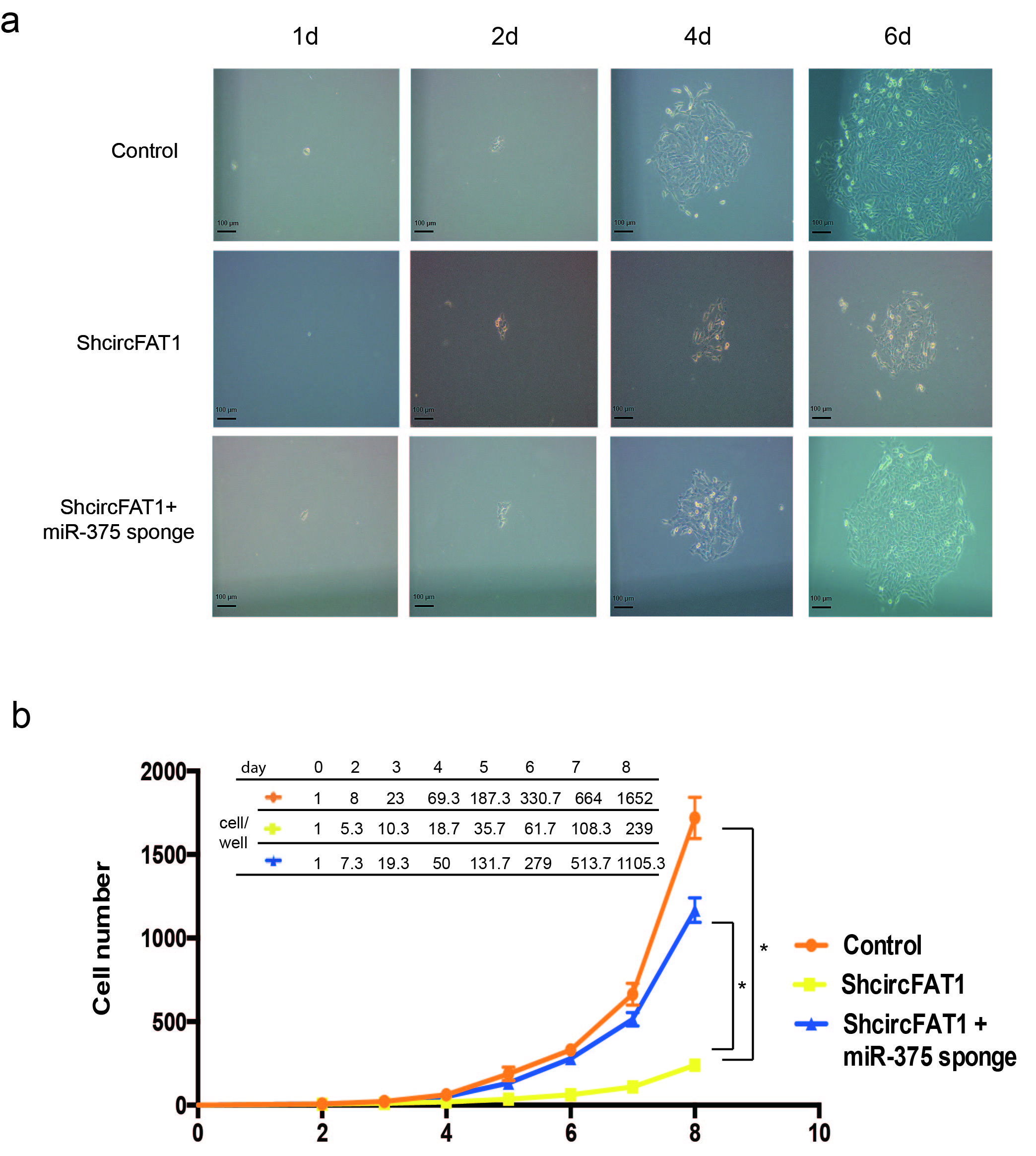

Supplement: Supplementary file 3 — Figure S7. Knockdown of miR-375 reverses shcircFAT1-induced attenuation of cell colony formation in OS cells. (a) 143B cells were inoculated in 96-well plates. Typical photos are shown for up to 6 days. Colony formation was monitored for up to 8 days. (b) Single-cell growth curve for up to 8 days. The numbers of cells per well are shown as the inset (Scale bar =100 μm). (JPG 1155 kb) [file 12943_2018_917_MOESM3_ESM.jpg]

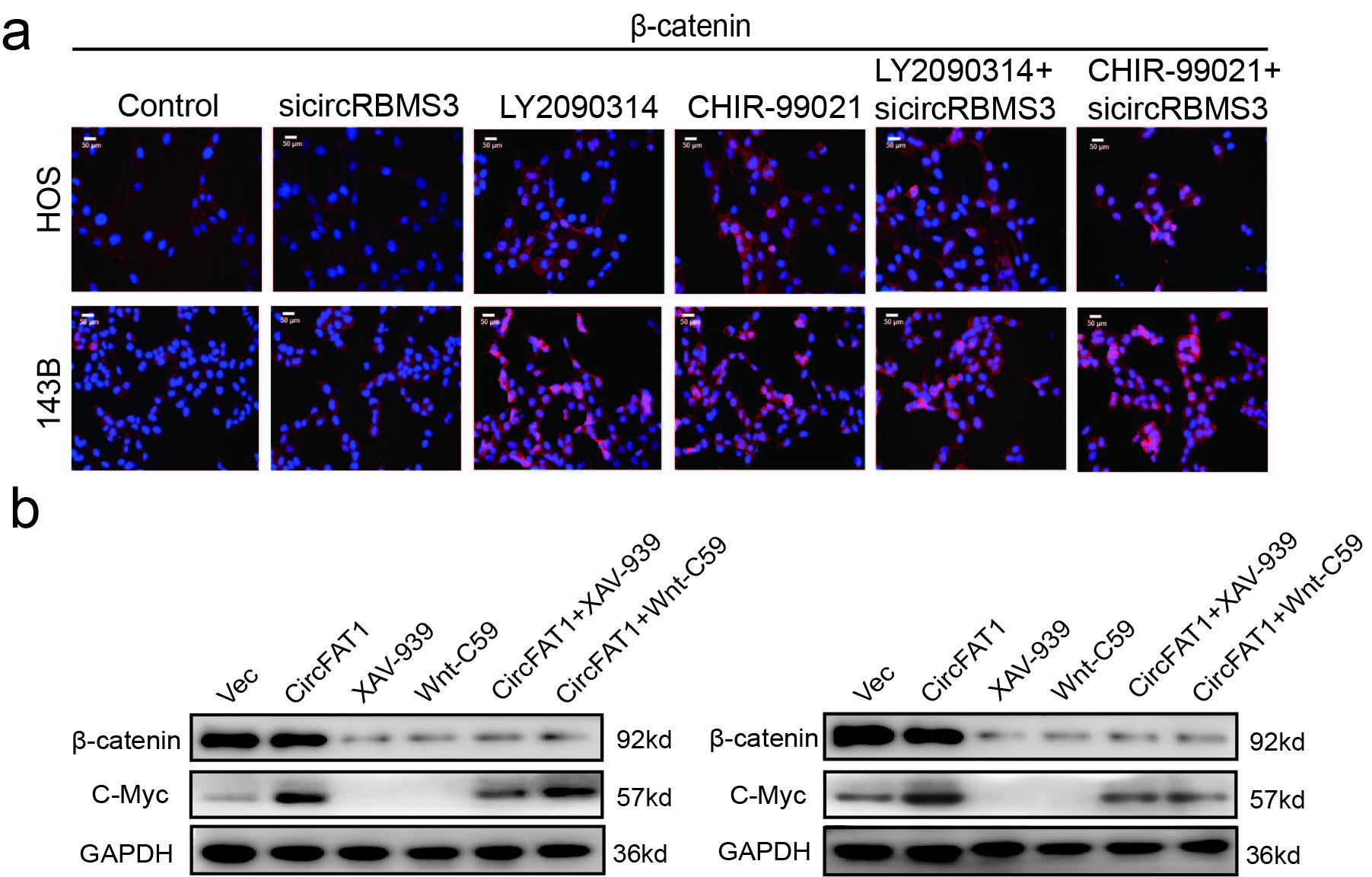

Supplement: Supplementary file 4 — Figure S5. The upregulation of c-Myc expression induced by circFAT1 overexpression is independent on Wnt signal pathway. (a) The β-catenin subcellular localization is detected by immunofluorescence analysis in OS cells with wnt activators' treatment. Scale bars = 50 μm. (b) The protein expressions of β-catenin and c-Myc in OS cells were detected by western blotting. Cells were co-transfected with circFAT1 or control vector, with or without wnt inhibitors. (JPG 1018 kb) [file 12943_2018_917_MOESM4_ESM.jpg]

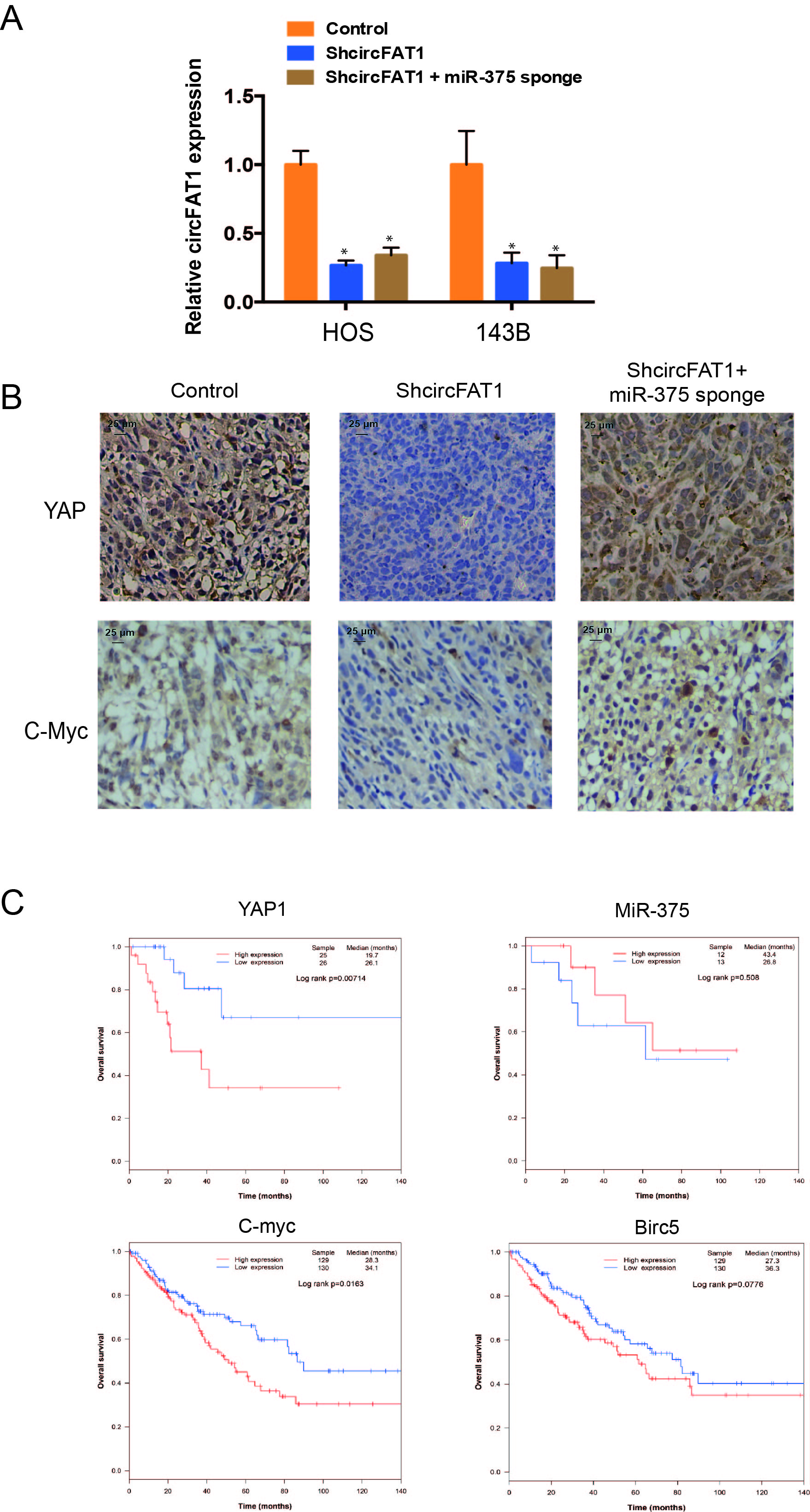

Supplement: Supplementary file 5 — Figure S8. CircFAT1 expression and Kaplan-Meier survival analysis. (a) QRT-PCR analysis of circFAT1 expression in tumors from xenograft mice. (b) The intra-nuclear localization of c-Myc and YAP. (c) Kaplan-Meier survival analysis of miR-375, YAP1, c-Myc and Birc5 low and high sarcoma patients (log rank test). (JPG 1414 kb) [file 12943_2018_917_MOESM5_ESM.jpg]

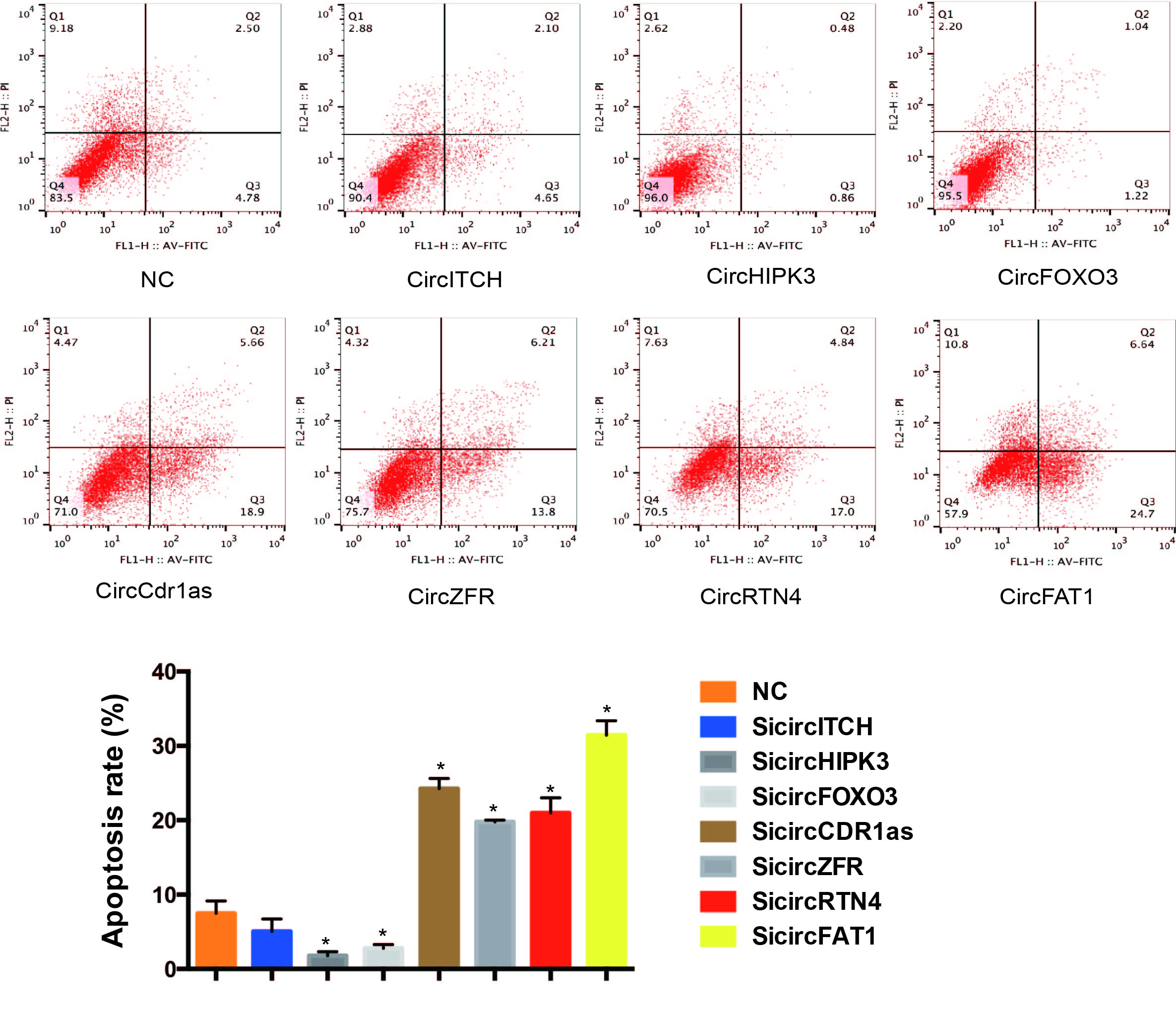

Supplement: Supplementary file 6 — Figure S1. The apoptosis rate of 143B cells with selected circRNAs knockdown. 143B cells were transfected with siRNAs of selected circRNAs for 48 h. Apoptosis rates were determined by Annexin V-FITC/PI staining. Data represent the mean ± SD (n = 3). * P < 0.05. (JPG 1341 kb) [file 12943_2018_917_MOESM6_ESM.jpg]
